# The critical role of KLF4 in regulating the activation of A1/A2 reactive astrocytes following ischemic stroke

**DOI:** 10.1186/s12974-023-02742-9

**Published:** 2023-02-23

**Authors:** Cong Wang, Longxuan Li

**Affiliations:** 1 Department of Neurology and Institute of Neurology, Ruijin Hospital, Shanghai Jiao Tong University School of Medicine, Shanghai, 200025 People’s Republic of China; 2 The Graduate School, Ningxia Medical University, Yinchuan, Ningxia 750004 People’s Republic of China

**Keywords:** Cerebral ischemic stroke, Kruppel-like transcription factor 4, Astrocyte, Inflammation

## Abstract

**Background:**

We have previously demonstrated that the expression of kruppel-like transcription factor-4 (KLF-4) is upregulated in astrocytes following acute ischemic stroke (AIS) and found that KLF4 confers vascular protection against cerebral ischemic injury. However, the functional role of KLF4 in astrocyte after AIS is far from clear.

**Methods:**

The intrinsic relationship between KLF4 and A1/A2 reactive astrocytes and the impact of astrocytic KLF4 on the activation of A1/A2 subtype astrocytes were evaluated in middle cerebral artery occlusion (MCAO) mice and oxygen–glucose deprivation and restoration (OGD/R) astrocytes.

**Results:**

Our results demonstrated that astrocytic KLF4 expression and complement C3-positive A1 and S100 calcium binding protein A10 (S100A10)-positive A2 astrocytes were induced in the ischemic penumbra following focal cerebral ischemia, and the time course of upregulation of astrocytic KLF4 correlated closely with the activation of A2 astrocytes. The dual immunofluorescent studies displayed that in the ischemic hemisphere, where the high levels of KLF4 were expressed, there were relatively low levels of C3 expressed in the reactive astrocytes and vice versa, but KLF4 was always co-stained well with S100A10. Mechanistic analyses revealed that astrocytic KLF4 inhibited the activation of A1 astrocyte but promoted A2 astrocyte polarization after OGD/R by modulating expressions of nuclear factor-kB.

**Conclusions:**

Astrocyte-derived KLF4 has a critical role in regulating the activation of A1/A2 reactive astrocytes following AIS.

## Introduction

Stroke, which includes ischemic stroke, is the second leading cause of death and disability worldwide. However, few effective treatments are currently available to the majority of stroke patients except for thrombolysis and endovascular thrombectomy, which are accessible to less than 10% of the patients [1]. Therefore, it is critical to develop effective prevention strategies.

Accumulating evidence indicates that neuroinflammation is involved in the pathogenesis of ischemic stroke [2, 3]. As a dominant non-neuronal cell population, astrocytes provide structural and functional support for neurons, release gliotransmitters to modulate synaptic activity, and participate in synapse formation and remodeling [4]. After the onset of stroke, astrocytes are immediately activated [5]. The classically activated astrocytes (A1 subtype) exert neurotoxic effects by releasing pro-inflammatory mediators, while alternatively activated astrocytes (A2 subtype) perform neuroprotective effects by secreting anti-inflammatory mediators [2, 6–8]. Thus, targeting the phenotypic transition of astrocytes (A1/A2 astrocytic polarization) is a promising strategy for the treatment of ischemic stroke.

Interestingly, immediately after the ischemic insult, many spontaneous protective mechanisms are activated to maintain cell homeostasis [9]. The kruppel-like transcription factor-4 (KLF-4) is an evolutionarily conserved zinc finger-containing transcription factor involved in a variety of cellular functions by activating or repressing the transcriptional activity of multiple genes [10]. Importantly, KLF4 is found to be up-regulated in the brain following ischemic injury [11, 12]. More recently, we found that the serum level of KLF4 is negatively correlated with infarct volume of acute ischemic stroke (AIS) patients and that KLF4 alleviates cerebral vascular injury by ameliorating vascular endothelial inflammation and regulating tight junction protein expression following ischemic stroke [13, 14], indicating that KLF4 confers vascular protection against cerebral ischemic injury. We also noticed that apart from brain endothelial cells and microglia, a large number of reactive astrocytes expressed KLF4 [13]. Nevertheless, the functional role of KLF4 in astrocyte after AIS is far from clear.

It is known that KLF4 suppresses the activation of inflammatory signaling [11, 13, 15]. we hypothesized that KLF4 may be a pivotal regulator of the functional phenotypes of activated astrocytes. Molecular screening studies have revealed that A1 and A2 subtypes of reactive astrocytes are detectably expressed in various CNS neurological disorders [6–8, 16]. Furthermore, complement C3 (C3) and S100 calcium binding protein A10 (S100A10) have been regarded as markers of A1 and A2 astrocytes, respectively [8]. In light of this, the aim of the current study was to investigate the temporal relationship between the expression of KLF4 and C3/S100A10 in astrocyte and uncover the functional role of KLF4 in regulating the phenotypes of activated astrocytes following AIS.

## Materials and methods

### Experimental animals

A total of 79 mice were used throughout this study. Forty-seven male C57BL/6 mice weighing 20–25 g (8–10 weeks of age) were used for middle cerebral artery occlusion (MCAO) surgery and 32 puppies within 48 h of birth were purchased from Shanghai Model Organisms Center, Inc (Shanghai, China) for primary astrocytic cultures. The present study was conducted in accordance with NIH guidelines for the care and use of animals in research and under protocols approved by the Animal Care and Use Committee of Ruijin Hospital, Shanghai Jiao Tong University School of Medicine.

### MCAO model

Focal cerebral ischemia was induced by reversible right MCAO surgery under pentobarbital anesthesia, followed by reperfusion as described previously [13, 17]. A laser-Doppler perfusion monitor (LDPM, PeriFlux5000, Perimed, Sweden) was used to measure the cerebral blood flow (CBF). The CBF was controlled by adjusting the filament in the artery for the induction of ischemia. Only the mice whose CBF displayed a drop of over 85% of baseline (before MCAO) just after MCAO were included for further experiment [18]. At the end of ischemia (90 min MCAO), mice were briefly re-anesthetized, and reperfusion was initiated by filament withdrawal. Relative CBF increased initially to 100% at 5 min post-reperfusion, and then stabilized at 70%. Sham animals (control) were subjected to the same procedure but did not receive MCAO. Mice were euthanized 0, 2, 4, 7, 14-day post-ischemia. The mean infarct volume in our hands was around 70 mm^3^ as previously reported by other research group [19] and the mortality rate of the mice was under 15%.

### Primary astrocytic cultures

Astrocyte culture was prepared as previously described [20–22]. Briefly, primary astrocytes were derived from the cortices of postnatal (P1 to P2) C57BL/6J mice. Cells were dissociated in Hank’s balanced salt solution (HBSS) containing 0.125% trypsin, followed by trituration. Subsequently, the cells were planted on T75 flasks in Dulbecco’s modified Eagle’s medium (DMEM) supplemented with 10% fetal bovine serum (FBS) and 1% penicillin/streptomycin, and then maintain in a humidified 5% CO_2_ incubator at 37 °C for 10–12 days. To purify astrocytes from microglia and oligodendrocytes, the cells in the T75 were subjected to continuous shaking at 37 °C (200 rpm, 16 h). The remaining cells were reseeded in 6- or 24-well culture plates for experiments, and a fraction of the cells were cultivated on chambered slides at 1 × 10^5^ cells per well to determine the purity of the astrocytes using immunofluorescence staining with anti-GFAP antibody. The purity of primary astrocyte cultures was approximately 95%.

### Construction and transfection of siRNA and pcDNA3.1 plasmids

Small interfering RNA (siRNA) and plasmids construction were performed as previously reported [13]. In brief, specific sequences of siRNA targeting the murine KLF4 (sense 5′-GGUCAUCAGUGUUAGCAAAGG-3′, antisense 5′-UUUGCUAACACUGAUGACCGA-3′) were designed and synthesized by Gene Pharma (Shanghai, China). Murine KLF4 (NM_010637.3) coding sequence was cloned into pCDNA3.1 plasmid vector (Invitrogen, Carlsbad, CA, USA) through EcoRI and XhoI sites. For cell transfection, confluent primary astrocytes were transfected with siRNAs or sequencing-verified constructs using Lipofectamine 3000 (Invitrogen) in accordance with the manufacturer’s protocol. After 48 h of transfection, the astrocytes were collected and used for further analysis. The control siRNA treated or mock-transfected astrocytes were used as negative control.

### Oxygen–glucose deprivation and restoration (OGD/R)

Oxygen–glucose deprivation and restoration (OGD/R) treatment was conducted according to a previously established protocol [22]. Forty-eight hours after transfection, primary astrocyte cultures were subjected to ischemia-like injury through OGD for 3 h by placing cultures in an anaerobic chamber (Forma, Thermo Scientific, Asheville, NC, USA) with an atmosphere of 5% CO2 and 95% N2 in a deoxygenated DMEM without glucose and FBS. After 3 h of OGD, cultures were returned to normal DMEM containing 10% FBS under normoxic conditions. Control cultures (no injury) were cultured with normal DMEM and 10% FBS for the same incubation times. All cultures were maintained in a humidified 37 °C incubator.

### Immunofluorescent staining and antibodies

Immunofluorescent (IF) staining was performed to examine how cerebral ischemia influences the expression of C3, S100A10 and KLF4 on astrocytes. Mice at different timepoints of reperfusion were euthanized by perfusion with ice-cold saline, and the brains were rapidly dissected and stored at − 80 °C. IF studies were performed as previously described on 10um thick frozen coronal sections [13, 14]. The following antibodies from Abcam (Cambridge, MA) were used in this study: rabbit anti-KLF4 polyclonal antibody (ab129473, 1: 500), chicken anti S100A10 polyclonal antibody (ab50737, 1:150), and rat anti-mouse Complement C3 monoclonal antibody (11H9, ab11862, 1:600). The Cy3-conjugated mouse anti-glial fibrillary acidic protein (GFAP) (clone G-A-5, 1:1500) was purchased from Sigma (St. Louis, MO, USA). The rabbit anti complement C3 polyclonal antibody (PA5-21349, 1:300), Alexa Fluor 488-conjugated goat anti-rat, and Cy3-conjugated goat anti-rat secondary antibody were obtained from Invitrogen (Carlsbad, CA, USA). Alexa Fluor 488-conjugated goat anti-rabbit, donkey anti-chicken, and Cy3-conjugated goat anti-rabbit secondary antibody were obtained from Jackson Immunoresearch (West Grove, PA, USA). The non-specific IgG or IgY isotype was used as a negative control for staining, which must be the same species as primary antibody.

For cultured primary astrocytes, 48 h after restoration from OGD, the cells were fixed in 4% paraformaldehyde for 30 min and then blocked in 1% BSA for 1 h. Next, the primary astrocytes were incubated with primary antibodies against Complement C3 (PA5-21349, 1:200, Thermo Fisher Scientific), S100A10 (PA5-95505, 1:300, Thermo Fisher Scientific) or GFAP (13-0300, 1:500, Thermo Fisher Scientific) overnight at 4 °C. Subsequently, the cells were incubated with appropriate secondary fluorescent antibodies. Finally, cell nuclei underwent counterstaining by utilizing DAPI (4′, 6-Diamidino-2-Phenylindole, Dihydrochloride) staining. Images were captured using a confocal microscope (Leica, Wetzlar, Germany).

Quantification of the number of positive cells for the different antigens on brain sections was performed as previous reported [13, 14]. Briefly, images of the region of interest were acquired using a × 20 or × 40 objective on a Leica TCS SP5 II microscope to determine the number of positive events per field of view (FOV). A minimum of three serial brain sections per mouse were selected for analysis of each antigen and matched between mice, so that the approximate position of sections used for IF staining was equivalent between different experimental conditions. Three images were taken from the ischemic penumbra including cortex and striatum as well as ischemic core of each brain section and quantified by eye for the number of positive events per FOV. The number of antigen-positive events per FOV for each section was calculated as the mean of total numbers obtained from the three regions. These averages of three brain sections were used for statistical analysis for each mouse.

### RNA extraction, reverse-transcription, and qPCR

Quantitative real time PCR (qPCR) analysis was used to determine the mRNA expression of IL-1β, IL-1ra, inducible nitric oxide synthase (iNOS), arginase1 (Arg1), tumor necrosis Factor (TNF)-α, and IL-10 in primary cultured astrocytes subjected to different treatments. Total RNA was extracted with Trizol reagent (Invitrogen, Carlsbad, CA) according to the manufacturer’s instructions. RNA was reverse transcribed into cDNA using a specific primer and a RevertAid First Strand cDNA Synthesis Kit (Thermo). qPCR was conducted using FastStart Universal SYBR Green Master (Rox) (Roche) and an ABI stepone-plus real-time PCR system. Forward and reverse primer sets for each cDNA were used as follows: 5′-AATGCCACCTTTTGACAGTGATG-3′ and 5′-GGAAGGTCCACGGGAAAGAC-3′ (for IL-1β, NM_008361.4); 5′-GTACTTACAAGGACCAAAT-3′ and 5′-TTCTCAGAGCGGATG-3′ (for IL-1ra, NM _001159562.1); 5′-ATGGCCTCCCTCTCATCAGT-3′ and 5′-TTTGCTACGACGTGGGCTAC-3′ (for TNF-α, NM _013693.3); 5′-GCCAAGCCTTATCG-3′ and 5′-GCATCCTGAGGGTCT-3′ (for IL-10, NM _010548.2); 5′-TGAAGAAAACCCCTTGTGCTG-3′ and 5′-CTCTCCACTGCCCCAGTTTT-3′ (for iNOS, NM _010927.4); 5′-AGGGTCCACCCTGACCTATG-3′ and 5′-TTCCCCAGGGTCTACGTCTC-3′ (for Arg1, NM _007482.3); and 5′-GGAGCGAGATCCCTCCAAAAT-3′ and 5′-GGCTGTTGTCATACTTCTCATGG-3′ (for GAPDH, NM _001289726.1). The average cycle threshold (Ct) value was normalized using the GAPDH signal. Relative transcript levels were calculated using the 2^−△△CT^ method. Each mRNA level was expressed as the fold-increase over the level of NO-OGD/R siRNA-Ctl or mock-treated group.

### Western blot analysis

Forty-eight hour after restoration of OGD, primary cultured astrocytes were harvested and lysed with lysis buffer (1% NP-40, 50 mM Tris HCl, pH 8.0, 150 mM sodium chloride) supplemented with protease and phosphatase inhibitor cocktails. Protein concentration was determined using the BCA protein assay kit (Eppendorf-Bio photometer, Germany). Western blotting and semi-quantitative analyses were performed as described previously [13, 14]. The following primary antibodies were purchased from Invitrogen (Carlsbad, CA, USA): rabbit anti-nuclear factor-κB (NF-κB) polyclonal antibody (51-3500, 1:1000), rabbit anti p-NF-κB polyclonal antibody (PA5-37658, 1:1000). Rabbit anti KLF4 polyclonal antibody (11880-1-AP, 1:1000) was gotten from Proteintech (Rosemont, IL). Rabbit anti S100A10 monoclonal antibody (ab232524, 1:1000), rabbit anti C3 monoclonal antibody (ab200999, 1:2000), rabbit anti TNF-α monoclonal antibody (ab215188, 1:1000), and rabbit anti iNOS polyclonal antibody (ab283655, 1:1000) were obtained from Abcam (Cambridge, MA). β-Actin were obtained from Neomarker (1:1000, Fremont, CA). Within each sample, the levels of proteins were first normalised to the level of β-actin, and then expressed as the fold-increase over the level of NO-OGD/R siRNA-Ctl or mock-treated group.

### Statistical analysis

All quantified data were expressed as mean ± standard deviation unless otherwise indicated. Statistical significance was assessed by one- or two-way analysis of variance (ANOVA), and a Bonferroni or Tukey’s post-hoc test was used to test multiple comparisons. All statistical analyses were performed using GraphPad Prism 8 software and significance was defined as *P* < 0.05.

## Results

### A1/A2 subtype reactive astrocytes and astrocytic KLF4 expression were induced in the ischemic penumbra following focal cerebral ischemia

Previous report showed that reactive astrocytes induced by lipopolysaccharide exhibited a phenotype suggesting that they may be detrimental, whereas reactive astrocytes in ischemia displayed a molecular phenotype implying that they may be beneficial or protective [23]. However, recent studies indicate that ischemic stroke can also induce two different polarized states of reactive astrocytes, termed neurotoxic A1 type and neuroprotective A2 type [7, 24]. Furthermore, C3 and S100A10 have been considered as markers for A1 and A2 astrocytes, respectively [8].

**Fig. 1:**
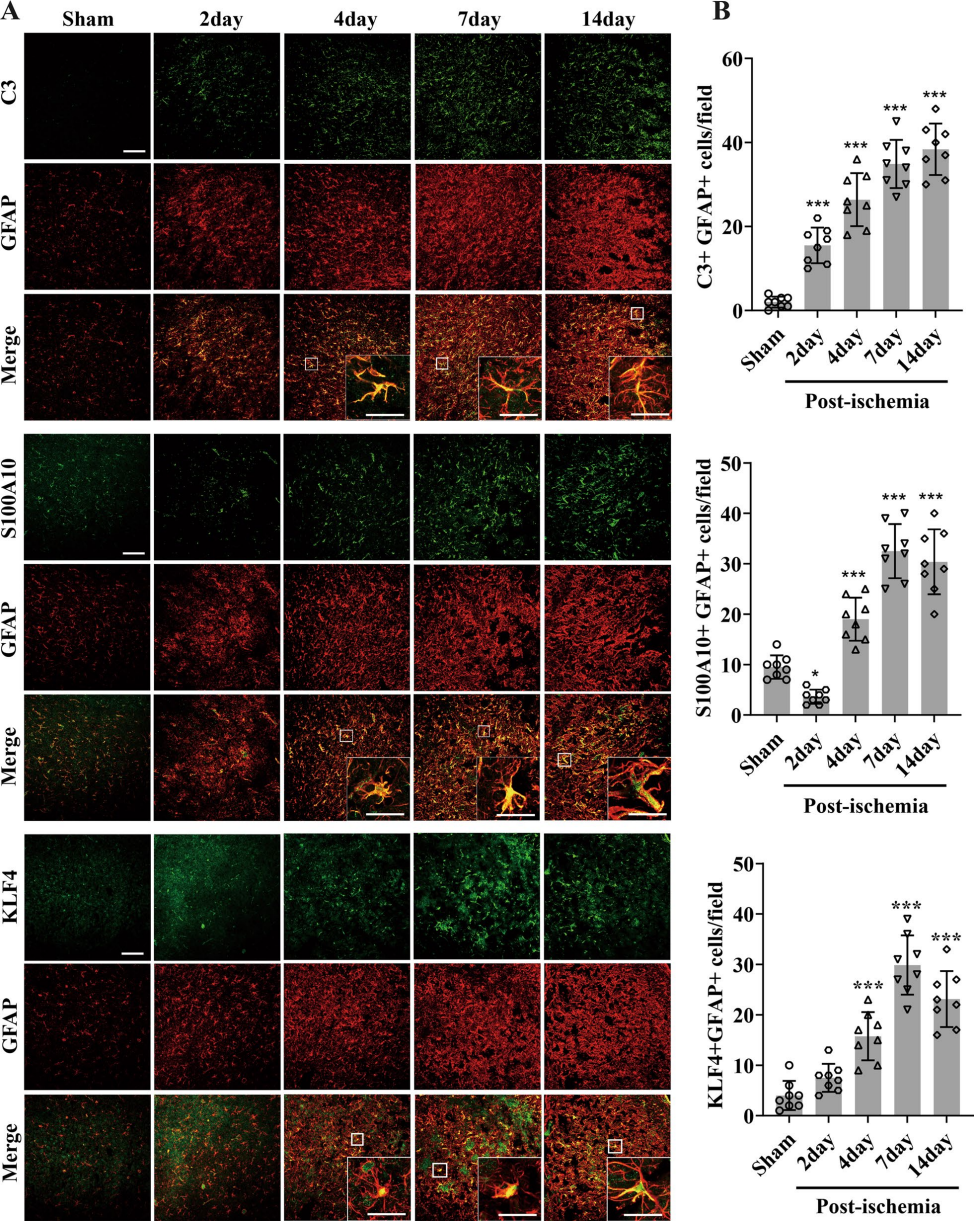
Activation of A1/A2 subtype astrocyte and expression of KLF4 on astrocyte following focal cerebral ischemia. **A** Images show the dual-IF staining for C3 with GFAP, S100A10 with GFAP, and KLF4 with GFAP in ischemic penumbra from sham operated mice (control) or mice at day 2, 4, 7 and 14 post-ischemia. Scale bar = 100 µm (inserts = 30 μm). **B** Quantification of A1-subtype (C3 + GFAP +) astrocyte, A2-subtype (S100A10 + GFAP +) astrocyte and KLF4 on astrocyte (KLF4 + GFAP +) expressions. Results are expressed as the mean ± standard deviation of the number of positive events per field of view, and the data were analyzed by one-way ANOVA (*n* = 8 per experimental group). Note that cerebral ischemia induced a strong increase in the number of A1 astrocytes in the penumbra, with the effect reaching maximal level at day 14 post-ischemia. However, the expression of A2 astrocyte decreased at day 2 post-ischemia, but then increased by 4–14-day post-ischemia, peaking at day 7, and declining at day 14. While the expression of KLF4 on astrocyte increased slightly during the first 2-day post-ischemia, but then increased significantly by day 4, and reached a maximum at day 7, before declining at day 14. *P < 0.05, ***P < 0.001 compared with control

In the current study, the dual-IF staining showed that, cerebral ischemia induced a strong increase in the number of A1 (C3 + GFAP +) subtype astrocyte in the penumbra, with the effect reaching maximal level at day 14 post-ischemia (Fig. 1A). Quantification revealed that compared with control brain (sham), the number of A1-subtype astrocytes/field in the penumbra at day 14 post-ischemia increased from 2 ± 1.31 to 38.38 ± 6.12 (*P* < 0.001) (Fig. 1B).

However, the number of A2 (S100A10 + GFAP +) astrocyte decreased at day 2 post-ischemia, but then increased by 4–14-day post-ischemia, peaking at day 7, and declining at day 14 (Fig. 1A). Quantification showed that compared with the control brain (sham), the number of A2-type astrocytes/field at day 7 post-ischemia increased from 9.5 ± 2.33 to 32.5 ± 5.37 (*P* < 0.001) (Fig. 1B).

The expression of KLF4 on astrocyte increased slightly during the first 2-day post-ischemia, but then increased significantly by day 4, and reached a maximum at day 7, before declining at day 14 (Fig. 1A). Compared with control brain, the number of KLF4 + GFAP + cells/field at day 7 increased from 4 ± 2.88 to 29.88 ± 5.87 (*P* < 0.001) in the penumbra (Fig. 1B).

### The relationship between expression of KLF4 and C3/S100A10 after cerebral ischemia

**Fig. 2:**
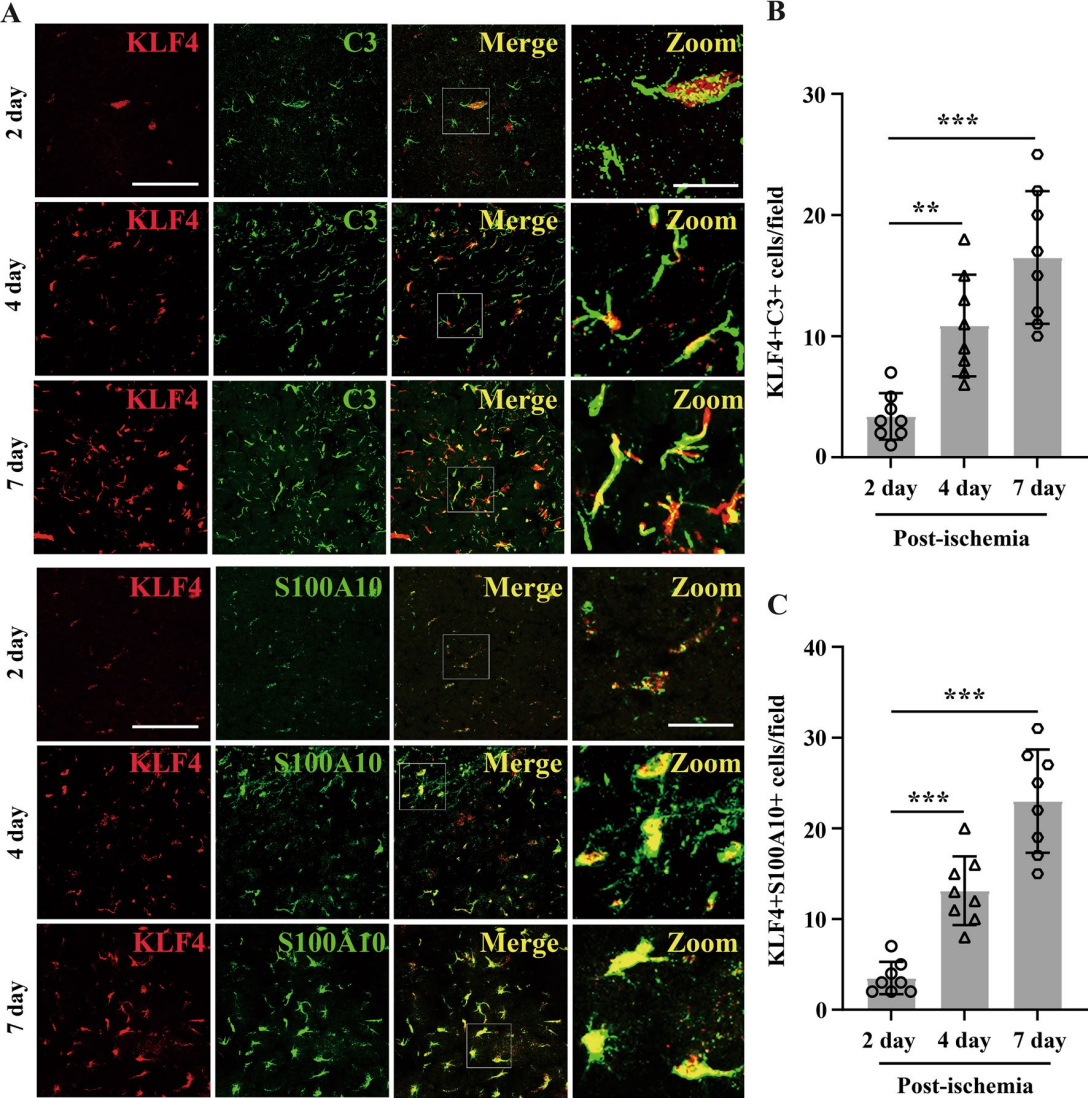
Co-expression of KLF4-C3 or KLF4-S100A10 after focal cerebral ischemia. **A** Images show the dual-IF staining for KLF4 with C3 as well as S100A10 in ischemic penumbra from mice 2, 4 and 7 days after focal cerebral ischemia. Scale bar = 100 µm for the left three columns, Scale bar = 25 µm for Zoom panels. **B**, **C** Quantification of KLF4 + C3 + cell number (**B**) and KLF4 + S100A10 + cell number (**C**) in the ischemic penumbra. Results are expressed as the mean ± standard deviation of the double-positive events per field of view, and the data were analyzed by one-way ANOVA (*n* = 8 per experimental group). Note that in the ischemic penumbra, the co-expression of KLF4-C3 or KLF4-S100A10 increased with time and reached a maximum level at day 7 post-ischemia. However, the distribution patterns of their expressions were different, where the high levels of KLF4 was expressed, relatively low levels of C3 were expressed in the ischemic penumbra, and vice versa. However, KLF4 was always co-stained well with S100A10 in the penumbra at days 2, 4 and 7 post-ischemia, especially at day 7, KLF4 co-localized with S100A10 extensively. **P < 0.01, ***P < 0.001 compared with 2-day post-ischemia

Based on the above findings that C3, S100A10 and KLF4 co-localized with GFAP, we next performed dual-IF to examine whether C3/S100A10 and KLF4 show any overlap in their expression profiles after cerebral ischemia. Of interest, as shown in Fig. 2A–C, in the ischemic penumbra, the co-expression of KLF4-C3 or KLF4-S100A10 increased with time and reached a maximum level at day 7 post-ischemia, whereas the distribution patterns of their expressions were different: where the high levels of KLF4 were expressed, there were relatively low levels of C3 expressed in the ischemic penumbra, and vice versa. However, KLF4 was always co-stained well with S100A10 in the penumbra at days 2, 4 and 7 post-ischemia, especially at day 7, KLF4 co-localized with S100A10 extensively. It seemed that the enhanced KLF4 could suppress A1 astrocyte expression of C3 but promote the activation of A2-type astrocyte after cerebral ischemia.

### KLF4 regulates the activation of A1/A2 subtype astrocyte following cerebral ischemia

In a recent study, we observed that KLF4 inhibited phosphorylation of NF-kB to alleviate the cerebral ischemia-induced cerebral vascular inflammation [13], and the latter was reported to be involved in the classical activation of astrocytes [25]. We wondered whether this mechanism also occurred in the reactive astrocyte following cerebral ischemia.

**Fig. 3:**
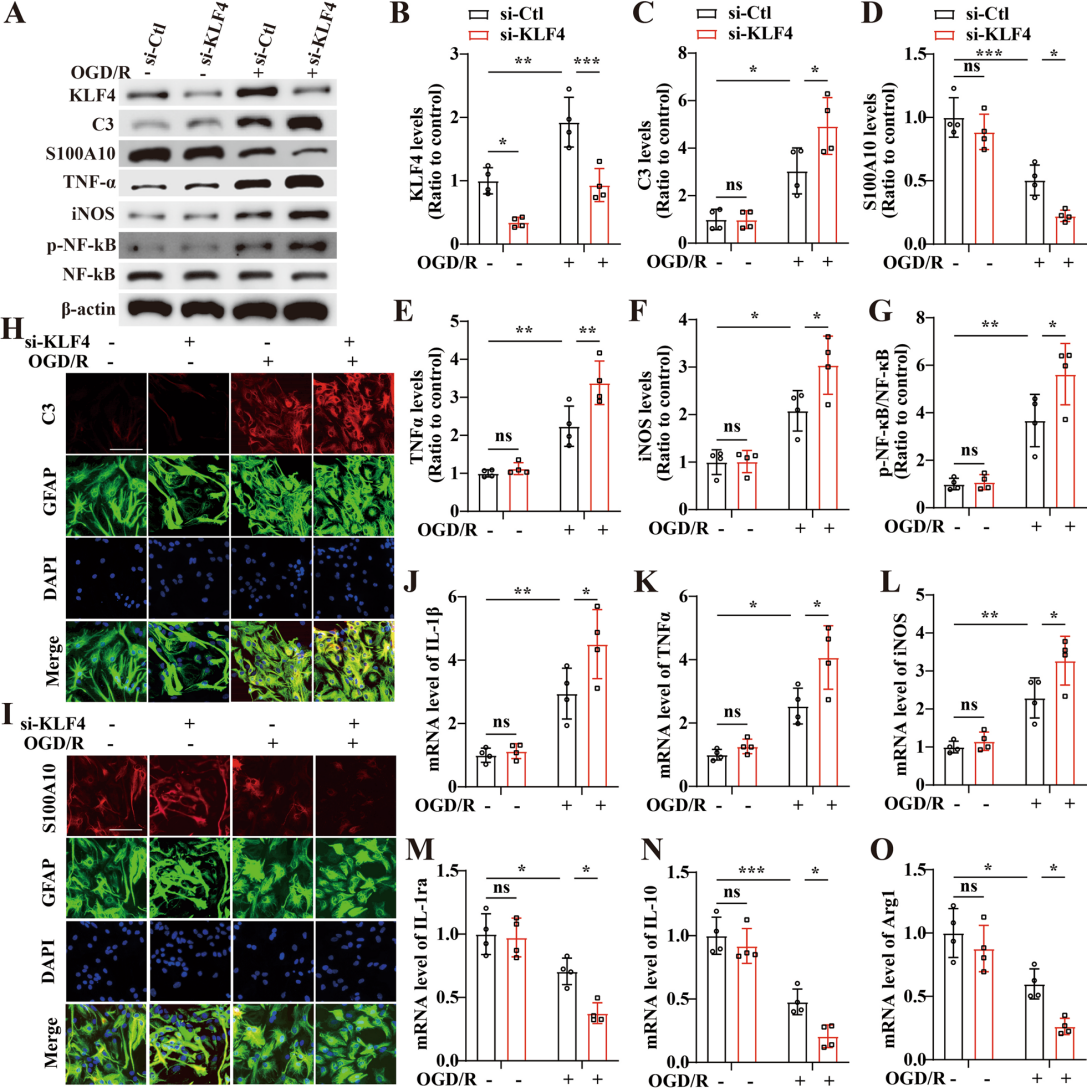
Influence of silencing of KLF4 on activation of A1/A2 astrocytes under OGD/R conditions. **A** Representative images of western blot for KLF4, C3, S100A10, TNF-α, iNOS, p-NF-kB in astrocytes transfected with the negative control siRNA (si-Ctl) or siRNA-KLF4 (si-KLF4) at 48 h restoration from OGD. The NO-OGD/R si-Ctl treated cells served as a control. **B**–**G** Bar graphs show the quantitative analyses of western blots as ratios of KLF4/β-actin (**B**), C3/β-actin (**C**), S100A10/β-actin (**D**), TNF-α/β-actin (**E**), iNOS/β-actin (**F**) and p-NF-kB/total NF-kB (**G**), and the data were analyzed by two-way ANOVA (*n* = 4 per experimental group). Note that the protein levels of C3, TNF-α, iNOS and the phosphorylation of NF-κB were significantly elevated relative to the si-Ctl-treated group at 48 h restoration from OGD, but the levels of KLF4 and S100A10 in the si-KLF4-treated astrocytes were markedly reduced. *P < 0.05, **P < 0.01, ***P < 0.001. **H**, **I** Representative images of immunofluorescent staining for C3/S100A10, GFAP, and DAPI in astrocytes after indicated treatments. Scale bar = 100 μm. **J**–**O** mRNA level of pro- or anti-inflammatory genes was determined by qPCR in the astrocytes of si-Ctl and si-KLF4 group at 48 h restoration of OGD or NO-OGD/R, and the data were analyzed by two-way ANOVA (*n* = 4 per experimental group). NO-OGD/R si-Ctl-treated cells served as control. Note that the mRNA levels of pro-inflammatory genes including IL-1β (**J**), TNF-α (**K**) and iNOS (**L**) were shown to significantly increase, but anti-inflammatory genes including IL-1ra (**M**), IL-10 (**N**), and Arg1 (**O**) were found to remarkably decrease in astrocytes after OGD/R injury. Furthermore, OGD/R-induced expression of pro-inflammatory genes was exaggerated by diminishing the expression of KLF4 in astrocytes; likewise, the decreased expression of anti-inflammatory genes caused by OGD/R was further augmented by silencing the levels of KLF4 in the astrocytes. *P < 0.05, **P < 0.01, ***P < 0.001; *ns* not significant

To confirm this, we employed KLF4-specific siRNA to knockdown the expression of KLF4 in primary astrocyte and performed western blotting to examine the impact of this on the expression of C3, S100A10, TNF-α, iNOS and phosphorylation of NF-kB in astrocyte in response to OGD/R. As shown in Fig. 3A–G, 48 h after restoration, the protein levels of C3, TNF-α, iNOS, and phosphorylation of NF-kB from the negative control siRNA (siRNA-Ctl)-treated astrocytes were significantly induced relative to the NO-OGD/R siRNA-Ctl-treated controls (*P* < 0.05 for C3;* P* < 0.01 for TNF-α; *P* < 0.05 for iNOS; *P* < 0.01 for phosphorylation of NF-kB); Furthermore, OGD/R-induced expression of these molecules and factors was enhanced by diminishing the level of KLF4 in the astrocytes (OGD/R siRNA-KLF4 vs. OGD/R siRNA-Ctl: *P* < 0.05 for C3;* P* < 0.01 for TNF-α; *P* < 0.05 for iNOS; *P* < 0.05 for phosphorylation of NF-kB). On the contrary, astrocyte expressions of S100A10 markedly decreased in response to OGD/R as compared to that of the NO-OGD/R siRNA-Ctl-treated controls (*P* < 0.001); Moreover, the decreased protein level of S100A10 caused by OGD/R was further augmented by silencing the level of KLF4 in the astrocytes (OGD/R siRNA-KLF4 vs. OGD/R siRNA-Ctl: *P* < 0.05). These data suggest that KLF4 regulates the A1/A2 activation of astrocytes following OGD/R.

**Fig. 4:**
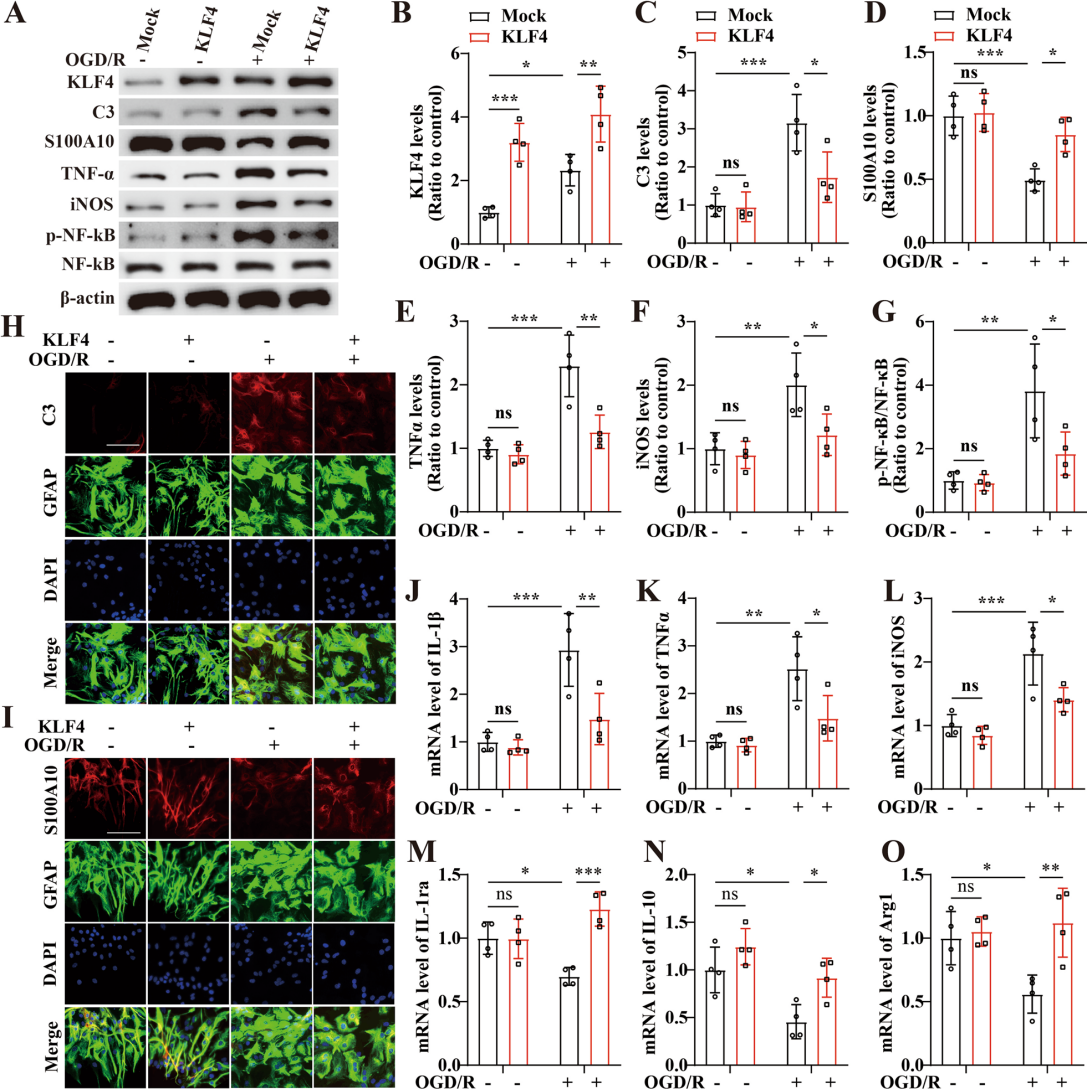
Impact of overexpression of KLF4 on activation of A1/A2 astrocytes under OGD/R conditions. **A** Representative images of western blot for KLF4, C3, S100A10, TNF-α, iNOS, p-NF-kB in astrocytes in astrocytes transfected with control plasmid (mock) or KLF4 overexpression plasmid at 48 h restoration from OGD. The NO-OGD/R mock treated cells served as a control. **B**–**G** Bar graphs show the quantitative analyses of western blots as ratios of KLF4/β-actin (**B**), C3/β-actin (**C**), S100A10/β-actin (**D**), TNF-α/β-actin (**E**), iNOS/β-actin (**F**), and p-NF-kB/total NF-kB (**G**), and the data were analyzed by two-way ANOVA (*n* = 4 per experimental group). Note that compared to the mock-transfected group, the protein levels of C3, TNF-α, iNOS and the phosphorylation of NF-κB were markedly reduced, but the levels of KLF4 and S100A10 were significantly elevated in astrocytes transfected with KLF4 after 48 h restoration from OGD. *P < 0.05, **P < 0.01, ***P < 0.001; ns, not significant. **H**, **I** Representative images of immunofluorescent staining for C3/S100A10, GFAP and DAPI in astrocytes transfected with mock or KLF4 overexpression plasmid after OGD/R treatment. Scale bar = 100 μm. **J**–**O** mRNA level of pro- or anti-inflammatory genes in the astrocytes were determined by qPCR in the mock or KLF4 overexpression group at 48 h restoration of OGD or NO-OGD/R, and the data were analyzed by two-way ANOVA (*n* = 4 per experimental group). NO-OGD/R mock-treated cells served as control. Note that compared to the mock-transfected group, overexpression of KLF4 decreased the expression of IL-1β (**J**), TNF-α (**K**), and iNOS (**L**), but increased the levels of IL-1ra (**M**), IL-10 (**N**), and Arg1 (**O**) in astrocytes under OGD/R conditions. *P < 0.05, **P < 0.01, ***P < 0.001; *ns* not significant

We then examined how overexpression of KLF4 altered astrocytic levels of C3, S100A10, TNF-α, iNOS and phosphorylation of NF-kB in response to OGD/R. Consistent with the observations in knockdown experiments, the western analysis revealed that 48 h after restoration, overexpression of KLF4 significantly inhibited the OGD/R-induced protein levels of C3, TNF-α, iNOS and the phosphorylation of NF-kB, but increased the level of S100A10 relative to the mock-treated group (Overexpression of KLF4 vs. Mock: *P* < 0.05 for C3;* P* < 0.01 for TNF-α; *P* < 0.05 for iNOS; *P* < 0.05 for S100A10; *P* < 0.05 for phosphorylation of NF-kB) (Fig. 4A–G).

The effects of KLF4 on astrocytic polarization were further validated by detecting the mRNA expression levels and immunocytochemistry of phenotypic markers. C3 and pro-inflammatory genes IL-1β, TNF-α, and iNOS were combined to mark neurotoxic (A1) astrocytes and S100A10 and anti-inflammatory genes IL-1ra, IL-10, Arg1 were combined to mark neuroprotective (A2) astrocytes [6, 8]. In the immunocytochemistry experiment, we found that inhibiting KLF4 led to an increased expression of C3 (Fig. 3H) but a decreased expression of S100A10 (Fig. 3I) in response to OGD/R. However, astrocytes transfected with KLF4 showed markedly reduced level of C3 (Fig. 4H), but higher level of S100A10 (Fig. 4I) relative to the mock-transfected group at 48 h restoration from OGD/R.

qPCR analyses demonstrated that the mRNA levels of pro-inflammatory genes including IL-1β, TNF-α and iNOS in astrocytes 48 h after OGD/R injury were significantly elevated, but anti-inflammatory genes including IL-1ra, IL-10 and Arg1 were remarkably reduced over the pre-OGD levels (*P* < 0.01 for IL-1β;* P* < 0.05 for TNF-α; *P* < 0.01 for iNOS;* P* < 0.05 for IL-1ra;* P* < 0.001 for IL-10*; P* < 0.05 for Arg1). Furthermore, OGD/R-induced expressions of pro-inflammatory genes were exaggerated by diminishing the expression of KLF4 in astrocytes (OGD/R si-KLF4 vs. OGD/R si-Ctl: all *P* < 0.05 for IL-1β, TNF-α and iNOS); likewise, the decreased expression of anti-inflammatory genes caused by OGD/R were further augmented by silencing the levels of KLF4 in the astrocytes (OGD/R si-KLF4 vs. OGD/R si-Ctl: all *P* < 0.05 for IL-1ra, IL-10 and Arg1) (Fig. 3J–O). However, in the overexpression experiments, transfection with KLF4 significantly decreased the expressions of pro-inflammatory genes but increased the levels of anti-inflammatory genes in the astrocytes relative to the mock-transfected group under OGD/R conditions (overexpression of KLF4 vs. mock: *P* < 0.01 for IL-1β;* P* < 0.05 for TNF-α; *P* < 0.05 for iNOS;* P* <0.001 for IL-1ra;* P* < 0.05 for IL-10*; P* < 0.01 for Arg1) (Fig. 4J–O). The above data indicated that KLF4 could inhibit neurotoxic polarization and promote neuroprotective polarization of astrocytes following cerebral ischemia.

## Discussion

In the current study, our main findings were as follows: (1) the C3-positive A1 astrocytes and expression of KLF4 on astrocyte were both induced in the ischemic penumbra following focal cerebral ischemia, but distribution patterns of their expressions were different, where the high levels of KLF4 was expressed, relatively low levels of C3 were expressed, and vice versa; (2) S100A10-positive A2 astrocytes increased in the ischemic penumbra, peaking at the same timepoint as KLF4 expression, and KLF4 was always co-stained well with S100A10; (3) KLF4 regulated the activation of A1/A2 subtype astrocytes after OGD/R. Taken together, we gave a clearer picture of the inter-relationship between astrocytic expression of KLF4 and the activation of A1/A2 reactive astrocytes.

### High expression of KLF4 is always associated with relatively less astrocytic expression of C3 in the ischemic hemisphere

KLF4 has been shown to be induced in reactive astrocytes following ischemic injury in vitro and in vivo [12]. More recently, we demonstrated that the serum level of KLF4 is negatively correlated with infarct volume at 48 h after ischemic onset in the AIS patients. Moreover, KLF4 can alleviate cerebral vascular injury by ameliorating vascular endothelial inflammation and regulating tight junction protein expression following ischemic stroke [13, 14]. These data indicate that KLF4 confers vascular protection against cerebral ischemic injury. However, the exact role of KLF4 in the reactive astrocytes after ischemia stroke remains poorly understood.

Evidence displayed that in macrophages, KLF4 controls their activation in response to lipopolysaccharide stimulation by regulating key inflammatory signaling pathways [10]. In light of this mechanism, we wondered whether KLF4 was also involved in regulating phenotypes of activated astrocytes following ischemic brain injury. To confirm this, in the current study, we used dual IF staining to reveal their intrinsic relationship between the expression of astrocytic C3 and KLF4 following ischemic stroke. We found that in response to cerebral ischemia, the expressions of C3 as well as KLF4 were both induced in GFAP positive astrocytes in the ischemic hemisphere over the 14-day time-course, but their distribution patterns were different: where the high levels of KLF4 were expressed, there were relatively low levels of C3, and vice versa. As KLF4 is a key factor in regulating inflammation [10], it seems likely that the enhanced KLF4 suppresses the astrocytic expression of C3 after cerebral ischemia.

### The time course of upregulation of astrocytic KLF4 correlates closely with the activation of A2 astrocytes following ischemic stroke

Some studies indicated that ischemia induced A2 astrocytic activation in vitro and in vivo, as evidenced by increased expression of S100A10 [15, 26]. However, other researchers reported that neuroprotective markers such as S100A10 and Arg1 in astrocyte decreased after OGD/R exposure [6]. This inconsistency may be related to the different timing of assessment and the degree of ischemia employed. In the current study, we found that the expression of S100A10 in astrocyte decreased at day 2 post-ischemia, but then increased by 4–14-day post-ischemia, peaking at day 7, and declining at day 14, and this is almost identical to the dynamic changes of KLF4 in astrocytes following ischemic stroke. Moreover, KLF4 was always co-stained well with S100A10. These results demonstrate that a tight correlation exists between astrocytic KLF4 expression and activation of A2 astrocyte after ischemic stroke.

### Astrocytic KLF4 inhibits activation of A1 astrocyte but promotes A2 astrocyte polarization

Previous evidence suggests that NF-κB-activated astrocytes release C3 to aggravate brain damage in Alzheimer’s disease [24, 27]. Furthermore, NF-κB inhibition caused by a decrease in reactive oxygen species levels was reported to be responsible for glycogen mobilization-induced A1-like and A2-like astrocyte transformation after ischemic stroke [26]. In a recent study, we observed that KLF4 inhibited TNF-a-induced activation of NF-kB to alleviate the cerebral ischemia-induced cerebral vascular inflammation [13]. Thus, the KLF4-NF-κB axis is likely to orchestrate various phenotypes of activated astrocytes following AIS. In the current study, we found that in response to OGD/R, C3 and pro-inflammatory genes such as iNOS, TNF-α and IL-1β were significantly induced in reactive astrocytes, accompanied by marked elevation of the phosphorylation of NF-κB. Furthermore, OGD/R-induced expression of these molecules and factors were enhanced by diminishing the levels of KLF4 in the astrocytes, whereas it was remarkably reversed by overexpression of astrocytic KLF4. These results suggest that KLF4 suppresses the activation of A1 astrocytes after OGD/R by regulating the expression of phosphorylation of NF-κB. On the contrary, we further found that astrocytic expression levels of S100A10 and anti-inflammatory genes (A2 subtype) including Arg1, IL-1ra and IL-10 were markedly reduced in response to OGD/R. Of interest, the decreased expression of A2 markers caused by OGD/R was further augmented by knockdown of KLF4 expression in the astrocyte, but it was significantly rescued by overexpression of astrocytic KLF4. This evidence suggests that KLF4 promotes the activation of A2 astrocytes after OGD/R.

## Conclusions

**Fig. 5:**
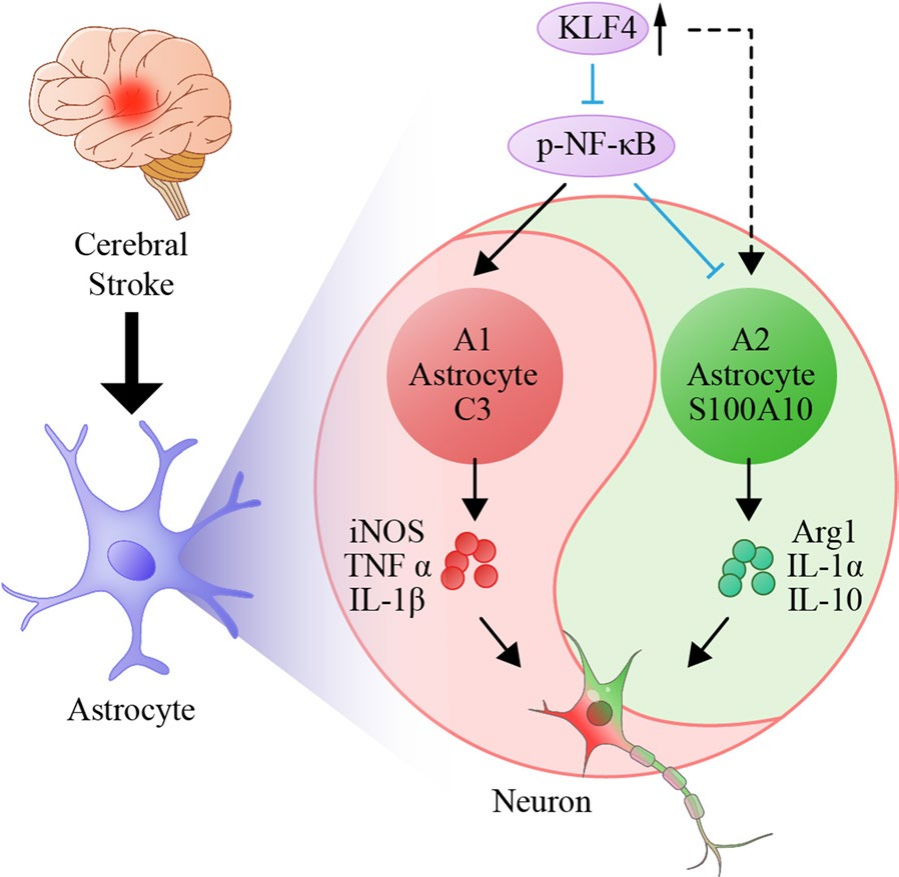
Schematic diagram showing astrocytic KLF4 regulating the activation of A1/A2 reactive astrocytes following ischemic stroke. After stroke onset, astrocytes are excessively activated. The classically activated astrocytes (A1 subtype) exert neurotoxic effects by releasing pro-inflammatory mediators, such as iNOS, TNF-α and IL-1β, while alternatively activated astrocytes (A2 subtype) perform neuroprotective effects by secreting anti-inflammatory mediators, such as Arg1, IL-1ra and IL-10. Of interest, astrocytic KLF4 inhibited activation of C3 positive A1 astrocytes but promoted S100A10 positive A2 astrocytes polarization following ischemic stroke by modulating expressions of nuclear factor-kB

Our results demonstrate cerebral ischemia induced activation of A1/A2 astrocytes and upregulated the expression of KLF4 in astrocytes. We also showed that astrocyte-derived KLF4 has a critical role in regulating the activation of A1/A2 reactive astrocytes following ischemic stroke by modulating expressions of NF-κB (Fig. 5). Nevertheless, further studies are needed to elucidate the molecular mechanisms by which KLF4 promotes A2 astrocyte polarization and to better understand the role played by KLF4 in this process.

## Data Availability

The data sets and materials supporting the conclusions of this article are included within the article.

## Abbreviations

AIS: Acute ischemic stroke
Arg1: Arginase1
IF: Immunofluorescent
iNOS: Inducible nitric oxide synthase
KLF 4: Kruppel-like transcription factor 4
MCAO: Middle cerebral artery occlusion
NF-κB: Nuclear factor-κB
siRNA: Small interfering RNA
S100A10: S100 calcium binding protein A10
TNF-α: Tumor necrosis factor

## References

[1] Aguiar de Sousa D, von Martial R, Abilleira S, Gattringer T, Kobayashi A, Gallofré M (2019). Access to and delivery of acute ischaemic stroke treatments: a survey of national scientific societies and stroke experts in 44 European countries. Eur Stroke J.

[2] Xu S, Lu J, Shao A, Zhang JH, Zhang J (2020). Glial cells: role of the immune response in ischemic stroke. Front Immunol.

[3] Xu Q, Zhao B, Ye Y, Li Y, Zhang Y, Xiong X (2021). Relevant mediators involved in and therapies targeting the inflammatory response induced by activation of the NLRP3 inflammasome in ischemic stroke. J Neuroinflammation.

[4] Perea G, Navarrete M, Araque A (2009). Tripartite synapses: astrocytes process and control synaptic information. Trends Neurosci.

[5] Amantea D, Micieli G, Tassorelli C, Cuartero MI, Ballesteros I, Certo M (2015). Rational modulation of the innate immune system for neuroprotection in ischemic stroke. Front Neurosci.

[6] Hong Y, Liu Q, Peng M, Bai M, Li J, Sun R (2020). High-frequency repetitive transcranial magnetic stimulation improves functional recovery by inhibiting neurotoxic polarization of astrocytes in ischemic rats. J Neuroinflammation.

[7] Rakers C, Schleif M, Blank N, Matušková H, Ulas T, Händler K (2019). Stroke target identification guided by astrocyte transcriptome analysis. Glia.

[8] Liddelow SA, Guttenplan KA, Clarke LE, Bennett FC, Bohlen CJ, Schirmer L (2017). Neurotoxic reactive astrocytes are induced by activated microglia. Nature.

[9] Tang W, Dong W, Xie P, Cheng P, Bai S, Ren Y (2015). The effect of pre-condition cerebella fastigial nucleus electrical stimulation within and beyond the time window of thrombolytic on ischemic stroke in the rats. PLoS ONE.

[10] Ghaleb AM, Yang VW (2017). Krüppel-like factor 4 (KLF4): what we currently know. Gene.

[11] Yang H, Xi X, Zhao B, Su Z, Wang Z (2018). KLF4 protects brain microvascular endothelial cells from ischemic stroke induced apoptosis by transcriptionally activating MALAT1. Biochem Biophys Res Commun.

[12] Park JH, Riew TR, Shin YJ, Park JM, Cho JM, Lee MY (2014). Induction of Krüppel-like factor 4 expression in reactive astrocytes following ischemic injury in vitro and in vivo. Histochem Cell Biol.

[13] Zhang X, Wang L, Han Z, Dong J, Pang D, Fu Y (2020). KLF4 alleviates cerebral vascular injury by ameliorating vascular endothelial inflammation and regulating tight junction protein expression following ischemic stroke. J Neuroinflammation.

[14] Wang C, Dong J, Sun J, Huang S, Wu F, Zhang X (2021). Silencing of lncRNA XIST impairs angiogenesis and exacerbates cerebral vascular injury after ischemic stroke. Mol Ther Nucleic Acids.

[15] El-Deeb NK, El-Tanbouly DM, Khattab MA, El-Yamany MF, Mohamed AF (2022). Crosstalk between PI3K/AKT/KLF4 signaling and microglia M1/M2 polarization as a novel mechanistic approach towards flibanserin repositioning in Parkinson’s disease. Int Immunopharmacol.

[16] Wang J, Sareddy GR, Lu Y, Pratap UP, Tang F, Greene KM (2020). Astrocyte-derived estrogen regulates reactive astrogliosis and is neuroprotective following ischemic brain injury. J Neurosci.

[17] Brait VH, Jackman KA, Walduck AK, Selemidis S, Diep H, Mast AE (2010). Mechanisms contributing to cerebral infarct size after stroke: gender, reperfusion, T lymphocytes, and Nox2-derived superoxide. J Cereb Blood Flow Metab.

[18] Liu F, Schafer DP, McCullough LD (2009). TTC, fluoro-Jade B and NeuN staining confirm evolving phases of infarction induced by middle cerebral artery occlusion. J Neurosci Methods.

[19] Li HD, Li M, Shi E, Jin WN, Wood K, Gonzales R (2017). A translocator protein 18 kDa agonist protects against cerebral ischemia/reperfusion injury. J Neuroinflammation.

[20] Hung CC, Lee YH, Kuo YM, Hsu PC, Tsay HJ, Hsu YT (2019). Soluble epoxide hydrolase modulates immune responses in activated astrocytes involving regulation of STAT3 activity. J Neuroinflammation.

[21] Yang XL, Wang X, Shao L, Jiang GT, Min JW, Mei XY (2019). TRPV1 mediates astrocyte activation and interleukin-1β release induced by hypoxic ischemia (HI). J Neuroinflammation.

[22] Han B, Zhang Y, Zhang Y, Bai Y, Chen X, Huang R (2018). Novel insight into circular RNA HECTD1 in astrocyte activation via autophagy by targeting MIR142-TIPARP: implications for cerebral ischemic stroke. Autophagy.

[23] Zamanian JL, Xu L, Foo LC, Nouri N, Zhou L, Giffard RG (2012). Genomic analysis of reactive astrogliosis. J Neurosci.

[24] Liddelow SA, Barres BA (2017). Reactive astrocytes: production, function, and therapeutic potential. Immunity.

[25] Liu M, Xu Z, Wang L, Zhang L, Liu Y, Cao J (2020). Cottonseed oil alleviates ischemic stroke injury by inhibiting the inflammatory activation of microglia and astrocyte. J Neuroinflammation.

[26] Guo H, Fan Z, Wang S, Ma L, Wang J, Yu D (2021). Astrocytic A1/A2 paradigm participates in glycogen mobilization mediated neuroprotection on reperfusion injury after ischemic stroke. J Neuroinflammation.

[27] Lian H, Yang L, Cole A, Sun L, Chiang AC, Fowler SW (2015). NFκB-activated astroglial release of complement C3 compromises neuronal morphology and function associated with Alzheimer’s disease. Neuron.

